# Incarerated femoral hernia in women – A critical view on approach options

**DOI:** 10.1016/j.ijscr.2023.108149

**Published:** 2023-04-12

**Authors:** Laura Pietrogiovanna, Joanna Janczak, Nina Pfeifer, Raphael Strahm, Walter Brunner

**Affiliations:** Department of General Visceral and Transplantation Surgery, Kantonsspital St. Gallen, Rorschacherstrasse, 95 9007 St. Gallen, Switzerland

**Keywords:** Femoral, Hernia, TAPP, Incarcerated, Strangulation, Inguinal, Mesh, Contamination

## Abstract

**Introduction and importance:**

In the literature there is few information on femoral hernias while best surgical approach to groin hernia in women is in recent discussion ([1], [2]). Focused on femoral hernia our purpose is to present a possible pathway for incarcerated female hernia approach demonstrated on four cases.

**Case presentation:**

Four female patients (77–90 y) with suspected incarcerated inguinal unilateral hernia undergoing repair at our department between December 2017 and December 2018 are presented. In three patients emergency laparoscopy by single port approach confirmed incarceration. Bowel was reduced and femoral hernia diagnosed. A TAPP repair was performed. The fourth patient had multiple previous abdominal operations due to anal carcinoma, so laparoscopic approach was not recommended. A transinguinal open approach also showed an incarcerated femoral hernia.

**Clinical discussion:**

In case of suspected incarcerated inguinal hernia accurate identification of a femoral hernia is necessary especially in female elderly patients. If possible endoscopic approach is preferred and offers exploration of both sides, checking bowel for vitality and fixing the hernia. If bilateral hernia is present, both sides should be addressed. Surgeons not used to TAPP should perform diagnostic laparoscopy with reduction of hernia sac and check of content and switch to TEP if experienced or open procedure. If open approach is necessary checking for femoral hernia is also mandatory and preperitoneal mesh placement is recommended with or without ligation of inferior epigastric vessels.

**Conclusion:**

Femoral hernias in women are not rare and in open repair techniques easily overseen. The endoscopic approach is preferred. With open approach the exploration via transversalis fascia is mandatory.

## Highlights

• In the literature is the treatment of femoral hernias in discussion.
• Importance of accurate identification of femoral hernias especially in female elderly patients.
• Incarceration and strangulation are the most common serious complications of femoral hernias.
• Endoscopic approach is the recommended option. If open approach the exploration via transversalis fascia is mandatory.

## 1 Introduction

Elective and emergent femoral hernia repairs constitute roughly 2–4 % of all groin hernia repairs and occur approximately 4 times more commonly in women, especially over the age of 50 (Little Old Ladies Hernia). Bilateral hernias are common and often the same subtype of hernia [3]. Clinical signs of femoral hernia are not always obvious and up to 40 % are overseen in open repair techniques [2], [4]. Furthermore, the question of repair in incidental finding on the contralateral side while incarceration repair is unanswered, as well as use of mesh in possible contamination due to incarceration.

The work has been reported according to the PROCESS criteria [11] and the SCARE 2020 criteria [12]. This is done to increase the robustness and transparency of this article.

## 2 Presentation of cases

Between 122,017 and 122,018 four female patients (77–90 y) were admitted with typical clinical signs of inguinal swelling, vomiting and abdominal pain and high suspicion of incarcerated unilateral femoral hernia (Sonography and CT) at our department.

Three patients had a primary hernia, one had a recurrence after Lichtenstein repair two years before for M2 hernia (medial hernia, size 1,5–3 cm) and a known contralateral inguinal hernia since four months which she refused to operate.

**Fig. 1:**
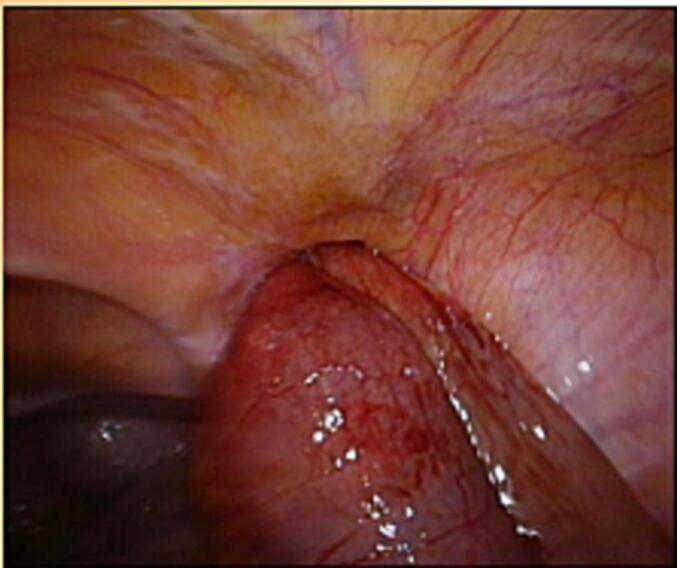
Incarcerated femoral hernia.

**Fig. 2:**
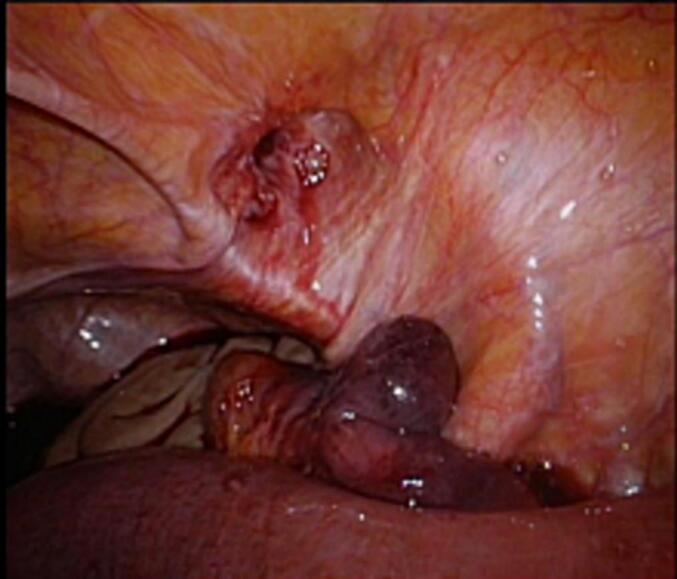
After bowel reduction.

**Fig. 3:**
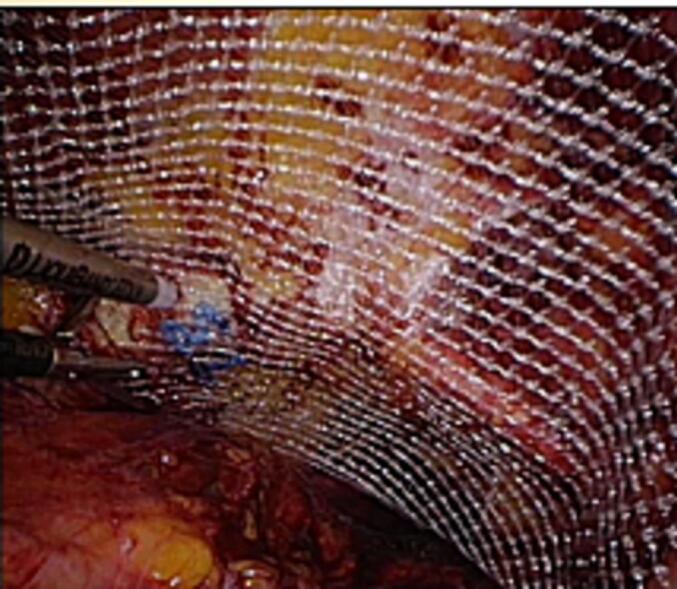
TAPP repair with 12 X 18 cm macroporous mesh.

Three patients had no previous abdominal operations. Therefore, emergency laparoscopy was performed. We choose an open transumbilical single port approach due to bowel distension. Bowel and omentum reduction was performed and a TAPP repair with 12 × 18 cm macroporous mesh was performed (Fig. 1, Fig. 2, Fig. 3). Round ligament was divided close to peritoneum for optimal mesh positioning and femoral overlap.

Patient 1: (Mb. Parkinson, 77 yo) had uneventful course after preferred method of repair.

Patient 2: (90 yo) with recurrence after Lichtenstein, which is another reason for posterior approach, underwent bowel resection due to necrosis. As extraperitoneal space was not contaminated, mesh placement was performed. The known contralateral side also was visualized and proved as femoral hernia, without repair in emergency situation and earlier refusal of patient.

Two days after uneventful course and discharge on day 8 she was readmitted unfortunately with contralateral incarceration and received TAPP repair after omentum reduction.

Patient 3: (79 yo) incidental finding of bilateral femoral and inguinal hernia and therefore bilateral TAPP repair was performed.

Patient 4: (80 yo) had multiple previous abdominal operations and radiochemotherapy due to anal carcinoma including mesh augmented stoma formation. Open approach was chosen as best approach and showed incarcerated femoral hernia. Due to massive adhesions inferior epigastric vessels had to be ligated, bowel was reduced. After peritoneal closure a preperitoneal mesh placement was performed with suture fixation to Coopers and inguinal ligament.

Uneventful further course for all patients, discharge between day 2 and 8, no recurrence occurred within 12 months' follow-up.

## 3 Discussion

Our cases show four different scenarios where all femoral hernias were diagnosed intraoperatively, as incarceration increases the challenge of preoperative differentiation from inguinal hernia. Surgical intervention remains the only option, best as tailored approach to situation and capabilities of local surgical facilities.

We show the importance of accurate identification of femoral hernias especially in female elderly patients. Laparoscopic approach therefore is the preferred and recommended option, offering exploration of both sides and all hernia defects, enabling visualisation of content and bowel for vitality and addressing the hernia. Furthermore, recurrence rates for groin hernia in women are less in endoscopic approaches, maybe due to overseen femoral hernias in open approach [2], [5], [6]. Incarceration and strangulation are the most common serious complications of femoral hernias and relatively higher than in inguinal ones, therefore repair of asymptomatic incidentally found femoral hernia is also recommended. If bilateral femoral hernia is present, both sides should be addressed [3], [7].

Surgeons not used to TAPP should perform diagnostic laparoscopy in case of incarcerated hernia with reduction of hernia sac and check of content and switch to TEP if experienced or open procedure with preperitoneal mesh repair [2], [8], [9]. Due to possible bowel distension in incarceration open transumbilical approach with single port system is our preferred method, whereas Veress needle is not recommended. Bowel resection can be performed via the protection foil of the single port.

In case of contraindications for laparoscopy as previous especially pelvic operations and radiotherapy or cardiopulmonary reasons open inguinal approach is recommended [2], [10]. Checking for femoral hernia is always mandatory. Preperitoneal mesh placement is recommended. The ligation of inferior epigastric vessels is an option to enlarge access if needed.

As mesh placement is preferred in groin hernia repair this should be considered in all cases where extraperitoneal space is clean or clean contaminated, which is the most common situation even in incarcerated cases. If contamination cannot be avoided, suture repair or postponing the hernia repair is an option. The use of biological or synthetic resorbable meshes maybe an option, but is still under discussion [2], [10]. Round ligament should not be divided in open approach, but for us seems useful in posterior approach for better overlap of femoral region.

## 4 Conclusion

Femoral hernias in women are not rare. The diagnostic pitfalls and in open repair techniques easily overseen. Exploration via transversalis fascia or endoscopic approach is recommended. Incarcerated hernias should be approached by laparoscopy, contralateral femoral hernia is not uncommon and simultaneous repair should strongly be considered. Preperitoneal mesh placement is preferred and, in most cases, possible, even with bowel resections.

## Patient consent

Written informed consent was obtained from the patients for publication of this case report and accompanying images. A copy of the written consent is available for review by the Editor-in-Chief of this journal on request.

## Provenance and peer review

Not commissioned, externally peer-reviewed.

## Ethical approval

This case series is exempt from ethical approval in our institution.

## Funding

No external funding was received for this study.

## Author contribution

Laura Pietrogiovanna wrote the original article. Joanna Janczak, Nina Pfeifer, Raphael Strahm treated the patients and critically reviewed the article. Walter Brunner supervised and critically reviewed the article

## Guarantor

Laura Pietrogiovanna

## Research registration number

Not applicable.

## Conflict of interest statement

None of the authors declared any conflicts of interest.
